# Synergy of de-walled Ganoderma Lucidum spore powder (GLSP) on targeted therapy in advanced non-squamous non-small cell lung cancer with epidermal growth factor receptor (EGFR) mutant: protocol for a randomized, double-blind, placebo-controlled study

**DOI:** 10.1186/s12906-024-04416-2

**Published:** 2024-03-18

**Authors:** Tong-Tong Wu, Yu-Yi Chen, Zi-Chun Yuan, Guo-Wang Yang, Gan-Lin Zhang

**Affiliations:** 1 Department of Oncology, Beijing Hospital of Traditional Chinese Medicine, Capital Medical University, No. 23, Back Road of Art Gallery, Dongcheng District, Beijing, 100010 China; 2 Department of Oncology, Shunyi Hospital, Beijing Hospital of Traditional Chinese Medicine, Beijing, China

**Keywords:** Non-small cell lung cancer (NSCLC), Repercussions associated with targeted therapy, Ganoderma Lucidum spore powder (GLSP), Randomized controlled trial (RCT), Fatigue, Evaluation of clinical curative effect

## Abstract

**Background:**

Osimertinib is regarded as a promising third-generation epidermal growth factor receptor (EGFR) tyrosine kinase inhibitor (TKI) for advanced non-squamous non-small cell lung cancer (NSCLC) patients who developed T790M. However the adverse effects, primarily fatigue, remain an overwhelming deficiency of Osimertinib, hindering it from achieving adequate clinical efficacy for such NSCLC. Ganoderma lucidum has been used for thousands of years in China to combat fatigue, while Ganoderma Lucidum spores powder (GLSP) is the main active ingredient. The aim of this study is to investigate whether GLSP is sufficiently effective and safe in improving fatigue and synergizing with Osimertinib in non-squamous NSCLC patients with EGFR mutant.

**Method/design:**

A total of 140 participants will be randomly assigned to receive either de-walled GSLP or placebo for a duration of 56 days. The primary outcome measure is the fatigue score associated with EGFR-TKI adverse reactions at week 8, evaluated by the Chinese version of the European Organization for Research and Treatment of Cancer (EORTC) Quality of Life Questionnaire for Cancer Patients (QLQ-C30). Secondary outcomes include evaluation of treatment effectiveness, assessment of quality of life (QoL), and exploration of immune indicators and gut microbiota relationships. Following enrollment, visits are scheduled biweekly until week 12.

**Trial registration:**

China Clinical Trial Registry ChiCTR2300072786. Registrated on June 25, 2023.

## Introduction

According to GLOBOCAN 2020, mortality of advanced lung cancer already accounts for 18.0% of that from overall cancer, and has been on rise constantly [1], despite the progress in systemic anti-tumor therapy for partial control or alleviation [2]. Notably, the advances in targeted therapies, driven by progressive genotype segmentation, have led to a de-chemotherapy era of molecular phenotypic personalization, represented by the third-generation epidermal growth factor receptor (EGFR) tyrosine kinase inhibitors (TKIs) such as Osimertinib, which has improved response rate (RR) to approximately 80% and prolonged median progress-free survival (PFS) to 10 ${\sim}$ 14 months for the most common Ex19del with T790M [3, 4]. However, 79-88% of cases have suffered from adverse effects, whose toxicity led to multi-organs dysfunction and poor quality of life [5], disrupting the subsequent long-term use of this drug [6].

Fatigue is the most common adverse event reported by more than two-thirds of patients on Osimertinib [7, 8]. The significant correlation has been demonstrated between fatigue and worse overall survival (OS) in those with cancers, that probably attributed to the constant aggravation of fatigue as a result of clinical neglect and absence of invention, ultimately eroding the general state, and even leading to the termination of anti-tumor therapy [9, 10]. Currently, the principal therapies for fatigue include psychiatric medications, erythropoietin supplementation, and non-pharmacological methods such as exercise and psychotherapy [11]. Regrettably, these approaches are far from effective while lead to additional adverse events or induce malignancies [12]. Thus, it is imperative to optimize the efficacy of Osimertinib associated fatigue in non-squamous NSCLC patients with EGFR mutant.

Ganoderma lucidum spore powder (GLSP), one of the effective ingredients of traditional Chinese herb Ganoderma lucidum, is primarily known for its qi replenishing properties. GLSP has a variety of benefits including anti-inflammatory, antioxidant and immunomodulation [13], suggesting its anti-fatigue potential. Besides, GLSP can inhibit tumor progression, by regulating CD4^+^CD8^+^ T cell ratio and macrophage function, especially the expression of PD-1 in TIME.

Therefore, we organized a multi-center, randomized, double-blind, placebo-controlled study to evaluate the synergy of de-walled GLSP on third-generation EGFR-TKIs represented by Osimertinib in advanced NSCLC with EGFR mutant. The objective of the study is to identify the improvement of de-walled GLSP on Osimertinib induced fatigue and general quality of life. In addition, the study also addresses the objective response to Osimertinib, and its safety. Furthermore, we sought to clarify the underlying mechanism of GLSP on the host immunity through liquid biopsy and intestinal flora assay.

## Methods/design

### Study design

**Fig. 1:**
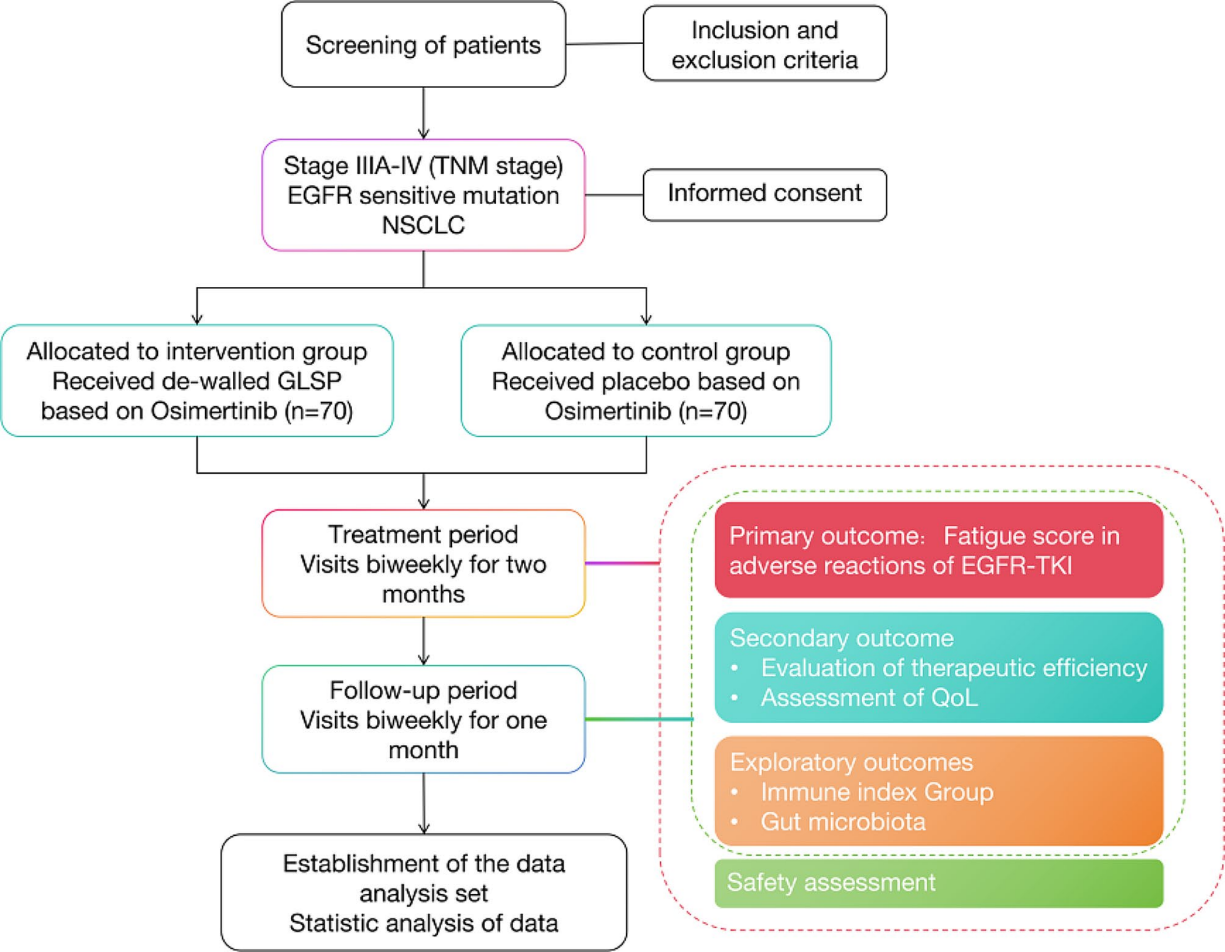
Study process / flow chart

This is a multicenter, randomized, double-blind, placebo-controlled clinical trial with significant advantages in its overall design. After a 7-day screening period, 140 eligible participants will be randomly assigned in a 1:1 ratio to either the trial group (de-walled GLSP + Osimertinib) or the control group (placebo + Osimertinib) through the Interactive Web Response System (IWRS). All participants will undergo two cycles of targeted and experimental drug therapy, followed by a one-month follow-up period. Visits are scheduled on the last day of every 14 days after enrollment. The study is scheduled to be open from February 2023 until February 2026. Recruitment will commence in August 2023 and last for a duration of 18 months (Fig. 1).

### Settings and participates

Participants will be recruited from thirteen general hospitals located in six different provinces in China, including Beijing Hospital of Traditional Chinese Medicine, Capital Medical University, China-Japan Friendship Hospital, Beijing Friendship Hospital, Capital Medical University, Beijing Chaoyang Hospital, Capital Medical University, Beijing Tongren Hospital, Capital Medical University, Henan Cancer Hospital, the First Affiliated Hospital of Hebei North University, and the First Central Hospital of Baoding City. Additionally, participants will also be recruited from The First Affiliated Hospital of Zhengzhou University, Hubei Provincial Hospital of Traditional Chinese Medicine, Gansu Provincial Cancer Hospital, Beijing Daxing District People’s Hospital and Ningbo Hospital of Traditional Chinese Medicine. Recruitment will be conducted through advertisements or notices posted on the websites and official WeChat accounts of these thirteen hospitals.

The inclusion criteria are as follows: (1) Non-squamous non-small cell lung cancer diagnosed by cytology/histopathology. (2) Clinical staging of inoperable stage IIIA-IV determined through imaging examinations, including chest-enhanced CT, head-enhanced CT/MRI, cervical/supraclavicular lymph node ultrasound/CT, upper abdomen-enhanced CT/B ultrasound, and whole-body bone scan. (3) Driver gene testing with retained tissue specimens after pathological diagnosis or peripheral blood free/tumor DNA (cf/ctDNA) if sufficient tissue specimens cannot be obtained is recommended for epidermal growth factor receptor (EGFR) gene mutations sensitive to 19DEL and 21L858R. (4) Lung lesions must be measurable. (5) ECOG-PS score should not exceed 2 and expected survival should be greater than 3 months. (6) The age range is from 18 to 75 years old. (7) Participants must agree to participate in this study and sign an informed consent form.

The exclusion criteria are as follows:(1) Presence of isolated brain/bone/adrenal metastases to be treated with radiation. (2) Pregnant or lactating women, or with cardiac, pulmonary, hepatic, renal, hematologic, or other systemic severities that are assessed by the investigator to be unsuitable for participation in this study. (3) Known allergy to the study drug. (4) The subject is participating in other clinical trials.

The withdrawal criteria are as follows: (1) Unexpected rapid progression or radiographic confirmation of progression. (2) Complications or coexisting conditions that prevent continued participation in the trial. (3) Serious adverse events occur that make continuation of the trial inappropriate. (4) Erroneous inclusion in the trial was identified. (5) Safety concerns are identified by the investigators. (6) Participants themselves decide to withdraw. Participants have the right to withdraw from the trial during the course of the trial, meanwhile investigators should attempt to identify and document reasons for withdrawal, such as a perceived lack of treatment effect. Some adverse reactions could not be tolerated, unexplained loss to follow-up, etc.

### Intervention

The programmatic interventions involve the utilization of Osimertinib, and de-walled GLSP.

#### Third-generation EGFR-TKI Osimertinib

The initial treatment regimen for EGFR-mutated NSCLC involves the administration of third-generation EGFR-TKI Osimertinib, as recommended by the NCCN Clinical Practice Guidelines for NSCLC 2022 V6. A targeted therapy duration of 56 days will be implemented during the study period.

#### De-walled Ganoderma Lucidum spore powder

All participants will commence administration on the first day of enrollment and continue for a duration of 2 months. The drugs should be completely dissolved by stirring at a temperature of 100 °C hot water, diluted with cold water or cooled prior to administration two grams twice per day during the initial week, and four grams twice per day from weeks 2 to 8. In parallel, the control group will receive placebo pellets composed of 95% dextrin and 5% de-walled GLSP following an identical protocol. Both TCM granules and placebos are uniformly manufactured by Zhejiang Shouxiangu Pharmaceutical Co., LTD (located at No.10 Shangcheng Road, Hushan Street, Wuyi County, Zhejiang Province) to ensure similarity in terms of appearance, smell, texture, and taste. Participants are required to return the kit along with its label to the CRC as evidence of timely medication usage for adherence evaluation.

### Randomization and allocation concealment

The IWRS of the ProResearchCom Intelligent Monitoring System for Clinical Research will be utilized. Upon enrollment of eligible participants, it is obligatory for the Clinical Research Coordinator (CRC) to input participant details into the IWRS. Once verified by either the Principal Investigators (PI) or Sub-Investigator (Sub-I), participants will be allocated to either the trial or control group in a 1:1 ratio. The IWRS will automatically generate unique identification numbers and drug codes for each participant, subsequently providing feedback on grouping results to the PI or sub-I, who will then notify the drug administrator regarding preparation of drugs corresponding to their respective assigned drug codes.

The current study utilizes a double-blind methodology, whereby the de-walled GLSP drinkable tablets and placebo are enclosed in visually indistinguishable cartons utilizing identical pouches. Each carton is allocated a drug number that conceals details regarding the participant’s group and type. Participants will remain unaware of their treatment allocation until the completion of the trial, while statistical analysts have access to complete group information but do not actively partake in the trial.

### Sample size calculation

The modeling calculations were conducted using the PASS 15.0 software, employing a Two-Sample t-test Assuming Equal Variance (bilateral test) with the following parameters: alpha = 0.05, power = 0.90, and N1:N2 = 1:1 ratio. The score of fatigue according to QLQ-C30 is the primary outcome measure of our study. In the FLAURA patient-reported outcome using the same scale, a mean fatigue score of 32.2 ± 24.9 was observed in patients with advanced EGFR-mutated NSCLC treated with Osimertinib [14]. Referring to previous literature, it is considered that the minimum clinically important difference (MID) in fatigue subscale is 11.1 and there exists a range of change between 11.1 and 22.2 points within this threshold value range for clinical significance assessment purposes [15]. The use of de-walled GLSP is assumed to result in at least a 15-point increase in fatigue score, indicating potential clinical significance for this drug intervention approach. By calculating N1 = N2 = 59 cases, we determined that the minimum required sample size would be 118 cases. Considering a dropout rate of 15%, adjusting N1 = N2 = 70 cases yields a minimum sample size requirement of 140 cases.

### Assessments and time-points

Assessments will be conducted biweekly from the beginning of the study until completion (Table 1).

**Table 1:** Trial visit schedule

Study stage		Baseline	Therapeutic period				Follow-up period	
Point of visit			1	2	3	4	5	6
Follow-up time points^a^		7 days before treatment	Day 14 of treatment	Day 28 of treatment	Day 42 of treatment	Day 56 of treatment	Day 14 after treatment	Day 28 after treatment
Time point (days)		-7 to 0	14	28	42	56	70	84
Visit windows (days)		± 3	± 3	± 3	± 3	± 3	± 7	± 7
Informed Consent		×						
Inclusion and exclusion criteria		×						
Demographic data		×						
History of diagnosis and treatment		×						
Blood and stool samples collected		×				×		
Laboratory and aided examination	Liver/ Renal/ Blood/ Urine/ Stoolfunction test	×				×		
	Twelve items of cytokines	×				×		
	Imageological examination	×			×			×
	ECG	×				×		
QLQ-C30/ PFS-CV/ EQ-5D		×	×	×	×	×		×
Evaluation of adverse reactions		×	×	×	×	×		×
AE&SAE		×	×	×	×	×		×
Compliance evaluation		×	×	×	×	×		×
Combination drug		×	×	×	×	×		×

*Abbreviations* ECG electrocardiograph, QLQ-C30 Quality of Life Questionnaire for Cancer Patients, PFS-CV Chinese version of the Piper Fatigue Scale, EQ-5D European Five Dimensional Health Scale, AE adverse events, SAE serious adverse event

^a^Patients will be followed up every two weeks

#### Primary outcome

Fatigue symptoms as adverse reactions to EGFR-TKIs is the primary outcome of this study, which will be evaluated by the EORTC QLQ-C30 Chinese version. The scale will be administered biweekly throughout the treatment period. The outcome endpoint will be determined based on the QLQ-C30 fatigue subscale score on day 56.

#### Secondary outcomes

The secondary outcomes are as follows:

The efficacy evaluation of the combination of de-walled GLSP and EGFR-TKIs will be evaluated based on objective response rate (ORR), disease control rate (DCR), and progression-free survival (PFS) according to RECIST 1.1 criteria. Imaging examinations and efficacy assessments will be conducted on day 0, day 42, and day 84 of the treatment period, calculating ORR/DCR, while PFS will be determined by follow-up at each treatment cycle during the follow-up period.

The QoL assessment will be conducted using the QLQ-C30, PFS-CV (Chinese version of the Piper Fatigue Scale), and EQ-5D (European Five Dimensional Health Scale). Participants will independently complete these three questionnaires with assistance from the investigator to evaluate patients’ QoL from a multidimensional perspective. The total score for QLQ-C30 is calculated by summing scores across domains and dividing by the number of items included, which is further linearly transformed using polarization. PFS-CV measures typical fatigue symptoms and is calculated as the mean sum of all topic scores, with subscale scores obtained by averaging four different dimensions separately. EQ-5D quantifies patient health status through five dimensions assessed via a utility index, while a visual scale allows direct scoring by patients on a scale of 0 to 100. QoL will be collected at baseline and each visit.

#### Exploratory outcomes

**Fig. 2:**
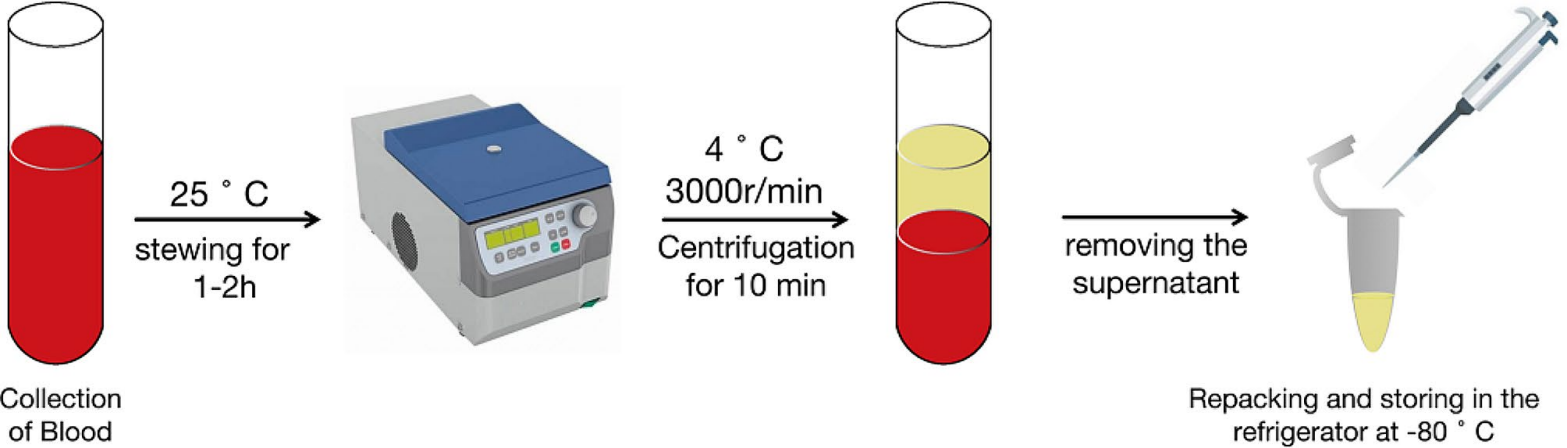
Blood sample collection

**Fig. 3:**
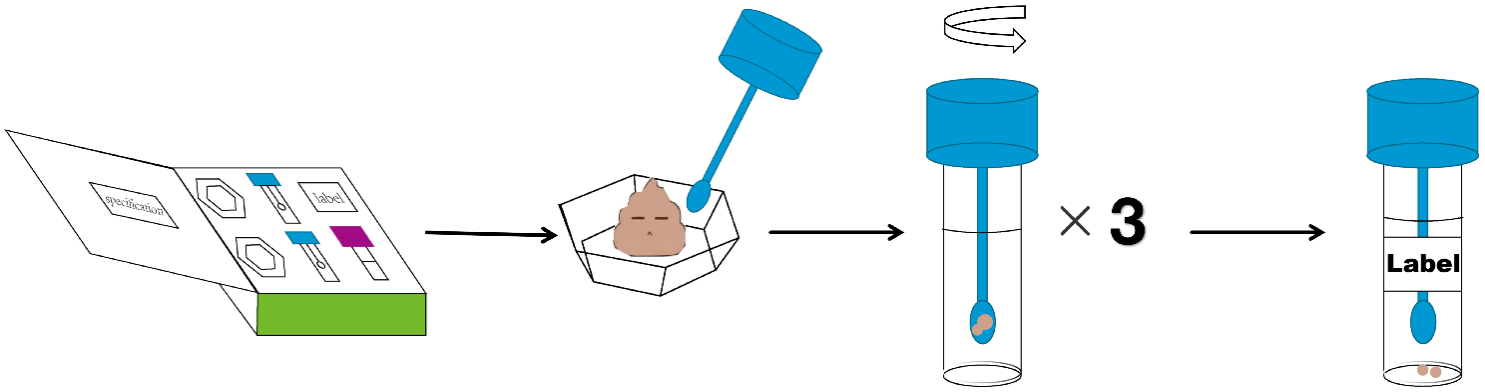
Stool sample collection

This study proposes exploratory indicators and recommends collecting blood for cytokine xii detection, such as IL-1β, IL-2, and TNF-α, and fecal samples for gut microbiota analysis to explore the subsequent mechanisms after obtaining informed consent from participants. All samples will be collected and transported to the central laboratory of Beijing Traditional Chinese Medicine Hospital for unified detection (Figs. 2 and 3).

#### Safety assessment

Participants will undergo regular monitoring of blood, urine, feces, liver and kidney function, as well as electrocardiograms on Day 0 and Day 28 of the treatment period. Adverse events will be classified according to the National Cancer Institute’s Common Terminology Criteria for Adverse Events (NCI-CTCAE), version 4.03. Any adverse events, serious adverse events, or adverse drug reactions occurring during the study will be documented with details including severity, relevance to this study, duration, relationship to the trial drug, management measures taken and outcome regression. Follow-up visits will continue until the patient’s condition stabilizes or improves.

### Register, ethical issues, and oversight

Before enrolling participants, we registered the study protocol with the China Clinical Trial Registry (ChiCTR2300072786). The study obtained ethical approval from the Ethics Committee of Beijing Hospital of Traditional Chinese Medicine, Capital Medical University (2023BL02-060-01) and other participating institutions. It is currently being conducted in accordance with the principles outlined in the Declaration of Helsinki regarding risks and benefits, privacy and confidentiality, informed consent, and placebo usage. Supervision will be carried out by Beijing Excellence Future International Pharmaceutical Technology Development Co., Ltd (CRO), with clinical research associates (CRAs) independently monitoring safety, quality, and progress on a quarterly basis.

### Data management

The responsibility for data management lies with Beijing Excellence Future International Pharmaceutical Technology Development Co., Ltd. The Data Manager (DM) of the company has implemented Electronic Data Capture (EDC) based on the Case Report Form (CRF) in ProResearch’s Clinical Research Intelligent Monitoring System, following the previously drafted Data Management and Validation Plan. Clinical Research Coordinators (CRCs) can input data into EDC using their accounts. Any errors or queries identified by the system will be forwarded to CRCs for resolution. Once all data entry issues have been resolved, PI, Sub-Is, and CRCs will lock the database within four weeks. Finally, Beijing Excellence Future International Pharmaceutical Technology Development Co., Ltd exports and transfers the locked database to statisticians for analysis purposes. Only the DM has access to the final trial dataset.

### Statistical analysis

The data will be subjected to two-sided statistical tests using SAS software (version 9.4). Statistical significance was defined as a P value of 0.05 or less. Following data collection, the Full Analysis set (FAS), per-protocol set (PPS), and safety set (SS) will be established. In order to be included in the per-protocol analysis, participants must provide data on the QLQ-LC30 at baseline and at least two additional time points. If a participant meets the withdrawal criteria, their data will not be included in the analysis. Continuous variables will be presented as mean ± SD or as median and IQR. Comparison between groups will be conducted using t-test or Wilcoxon rank sum test for continuous variables, while categorical variables will be expressed as frequency or proportion and compared using χ^2^ or Fisher’s exact test between groups. The survival analysis or Logistic model or Cox regression model will then be constructed for multivariate analysis based on these comparisons. Missing data will undergo multiple imputations to handle them appropriately, allowing for sensitivity analyses by comparing results from complete cases with those from imputed data.

## Discussion

Oncologic and survival outcomes are no longer the exclusive criteria for clinical benefit in patients with advanced NSCLC. Only if damages to QoL have been ruled out, will the superior outcomes be considered [16]. Although there is prolonged PFS and OS of Osimertinib over the earlier EGFR-TKIs, and a reduction in AE like elevated aminotransferase or rash, its prominent fatigue has become a tough nuisance exacerbating QoL concerns in patients with advanced NSCLC [14]. In fact, fatigue was ranked by 74% of patients as one of the most distressing symptoms associated with cancer related treatments, ahead of nausea and vomiting [17]. The lasting fatigue would certainly hinder the psychological health of patients and the maintenance their of social roles [18].

Numerous mechanisms of fatigue have been investigated over the past two decades, including dysregulation of the hypothalamic-pituitary-adrenal (HPA) axis, disorder of the 5-hydroxytryptamine (5-HT), and muscle metabolism changes induced by adenosine triphosphate (ATP), regulation of immunity by pro-inflammatory cytokine, etc. [19]. Effective reversal on any of the above pathways could be a breakthrough for Osimertinib induced fatigue. As shown in published studies, qi-replenishing herb has an obvious improvement on fatigue by rebalancing the immune microenvironment via regulating the levels of cytokines IL-1β, IL-6, IL-2 and IL-4 [20]. Concurrently, our unpublished data suggested that GLSP could affect host immunity by inhibiting thymocyte apoptosis, promoting macrophage polarization and phagocytosis, stimulating an increased ratio of CD3^+^CD8^+^ T cells, and modulating the secretion of chemokines and cytokines. On the basis, the process of separating the shells from the GLSP active ingredients has been optimized, which resulted in a more than 8-fold increase in polysaccharide and triterpenoid.

This is the first multicenter RCT, to our knowledge, to investigate GLSP on Osimertinib induced fatigue and its underlying immune mechanism. It does have some strengths in design as follows: (1) We select the patient-reported outcome (PRO) as our primary endpoint due to the subjective nature of fatigue, which is more suitable within the context of combination therapy for advanced NSCLC [21]; (2) the exploration of the possible mechanisms of TCM enhances the evidence-based value of this study and makes its use in modern clinical practice more objective. While lacking of powerful published data for sample size calculation is the main limitation of this study because of its pioneering character. Thus, the minimum important difference (MID) based on the GLSP effect was used in calculating the scale, with consideration of recruitment period and costs.

## Conclusion

In conclusion, the study results will provide powerful evidence for GLSP on Osimertinib induced fatigue.

## Data Availability

No datasets were generated or analysed during the current study.

## Declarations

### Trial status

Recruitment of subjects is currently underway. Recruitment began on September 1, 2023.
